# Left ventricular hypertrophy detection using electrocardiographic signal

**DOI:** 10.1038/s41598-023-28325-5

**Published:** 2023-02-13

**Authors:** Cheng-Wei Liu, Fu-Hsing Wu, Yu-Lun Hu, Ren-Hao Pan, Chuen-Horng Lin, Yung-Fu Chen, Guo-Shiang Tseng, Yung-Kuan Chan, Ching-Lin Wang

**Affiliations:** 1 Division of Cardiology, Department of Internal Medicine, Tri-Service General Hospital Songshan Branch, National Defense Medical Center, Taipei, Taiwan; 2 Bachelor Degree Program of Artificial Intelligence, National Taichung University of Science and Technology, Taichung, Taiwan; 3 Department of Management Information Systems, National Chung-Hsing University, Taichung, Taiwan; 4 La Vida Tec. Co. Ltd., Taichung, Taiwan; 5 Preventive Medicine Center, National Yang Ming Chiao Tung University, Taipei, Taiwan; 6 Department of Information Management, Tunghai University, Taichung, Taiwan; 7 Department of Computer Science and Information Engineering, National Taichung University of Science and Technology, Taichung, Taiwan; 8 Department of Dental Technology and Materials Science, Central Taiwan University of Science and Technology, Taichung, Taiwan; 9 Division of Cardiology, Department of Internal Medicine, Taoyuan Armed Force General Hospital Hsinchu Branch, Hsinchu, Taiwan; 10 Department of Information Management, National Chin-Yi University of Technology, Taichung, Taiwan

**Keywords:** Computational biology and bioinformatics, Cardiology

## Abstract

Left ventricular hypertrophy (LVH) indicates subclinical organ damage, associating with the incidence of cardiovascular diseases. From the medical perspective, electrocardiogram (ECG) is a low-cost, non-invasive, and easily reproducible tool that is often used as a preliminary diagnosis for the detection of heart disease. Nowadays, there are many criteria for assessing LVH by ECG. These criteria usually include that voltage combination of RS peaks in multi-lead ECG must be greater than one or more thresholds for diagnosis. We developed a system for detecting LVH using ECG signals by two steps: firstly, the R-peak and S-valley amplitudes of the 12-lead ECG were extracted to automatically obtain a total of 24 features and ECG beats of each case (LVH or non-LVH) were segmented; secondly, a back propagation neural network (BPN) was trained using a dataset with these features. Echocardiography (ECHO) was used as the gold standard for diagnosing LVH. The number of LVH cases (of a Taiwanese population) identified was 173. As each ECG sequence generally included 8 to 13 cycles (heartbeats) due to differences in heart rate, etc., we identified 1466 ECG cycles of LVH patients after beat segmentation. Results showed that our BPN model for detecting LVH reached the testing accuracy, precision, sensitivity, and specificity of 0.961, 0.958, 0.966 and 0.956, respectively. Detection performances of our BPN model, on the whole, outperform 7 methods using ECG criteria and many ECG-based artificial intelligence (AI) models reported previously for detecting LVH.

## Introduction

Left ventricular hypertrophy (LVH) is an early stage of structural heart disease associated with the incidence of cardiovascular diseases even mortality^1^. LVH contributes to subclinical end organ damage in hypertensive patients and is associated with poor prognosis of cardiovascular diseases^1–4^. LVH is currently defined using various methods, including electrocardiography (ECG), echocardiography (ECHO) and cardiac magnetic resonance imaging (cMRI)^5^. Detecting LVH using ECG is time efficient and reproducible. The diagnosis of LVH by ECG is made according to various criteria^6^, such as Cornell and Sokolow–Lyon criteria, which check the summations of voltage amplitudes of S and R waves representing the left ventricle depolarization^7,8^. Transthoracic echocardiography (TTE) directly measures the wall thicknesses of the left ventricular septum and posterior wall, but the measurement accuracy is dependent on physicians’ experiences of performing TTE^5^. The ECG examination features simple, reproducible and rapid, which is suitable to screen LVH in the general population, especially in largely healthy populations^9,10^.

Although ECG-based new criteria or new studies using existing criteria for detecting LVH have been proposed or reported in recent years, the overall sensitivity and precision are still disappointing^11–19^. These criteria mainly take the combination of multi-lead peak and valley amplitudes of R and S waves, associated with ventricular depolarization, as the threshold for diagnosing LVH. Artificial intelligence (AI) or machine learning models for detection LVH using the ECG with or without extra features (including demographics, anthropometric parameters, etc.) have also been proposed in recent years, however, the overall detection performances still need to be improved^20–27^.

Therefore, this study aims to develop a system for identifying LVH using our suggested ECG signal processing method and machine learning model to improve the LVH detection performances using ECG.

## Materials and methods

### Data

#### Study cohort

We retrospectively collected ECG and TTE data from a cohort registered in the division of Cardiology, Tri-service General Hospital Songshan Branch, Taipei, Taiwan between Jan. 1, 2016 and Dec. 31, 2017. Apparently healthy individuals with abnormal ECG findings at a health examination and who also underwent TTE were enrolled^9,10^. The time interval between the ECG and echocardiogram examinations in each case included in this study was within one to three months and the echocardiogram examination followed the ECG examination. The cohort consisted of 952 individuals, 856 men and 96 women, including 173 LVH cases (18%) and 779 non-LVH cases (82%). The dataset of this study was collected in the Tri-service General Hospital Songshan Branch which is a military hospital in Taipei, Taiwan. Many patients of this hospital are the military personnel and most military personnel are male in Taiwan. This explains the gender difference in this study. All methods were performed in accordance with the relevant guidelines and regulations. The ECG measurement was performed with the ECG machine, Philips Page Writer TC30, following recommendations of the American Heart Association for the standardization and interpretation of ECG. The TTE measurement was performed with Philips IE33 echocardiographic equipment according to contemporary guidelines^28,29^. The study was approved by the Institutional Review Board at Tri-Service General Hospital (No. TSGH 2-106-05-148), Taipei, Taiwan. This board judged that the informed consent was not necessary regarding the very low risk of the present study design. Our study was previously registered at ClinicalTrials.gov with the identification number NCT03473951^30^. Furthermore, data were deidentified and meet privacy rules of the health insurance portability and accountability act (HIPAA).

#### LVH definition

The standard LVH can be obtained from echocardiography by calculating the left ventricle mass index (LVMI) using the Devereux formula. And LVH meets LVMI greater than 115 g/m^2^ for a man and 95 g/m^2^ for a woman^19,28^. The definition was also used in our previous study^31^.

### System architecture

**Figure 1:**
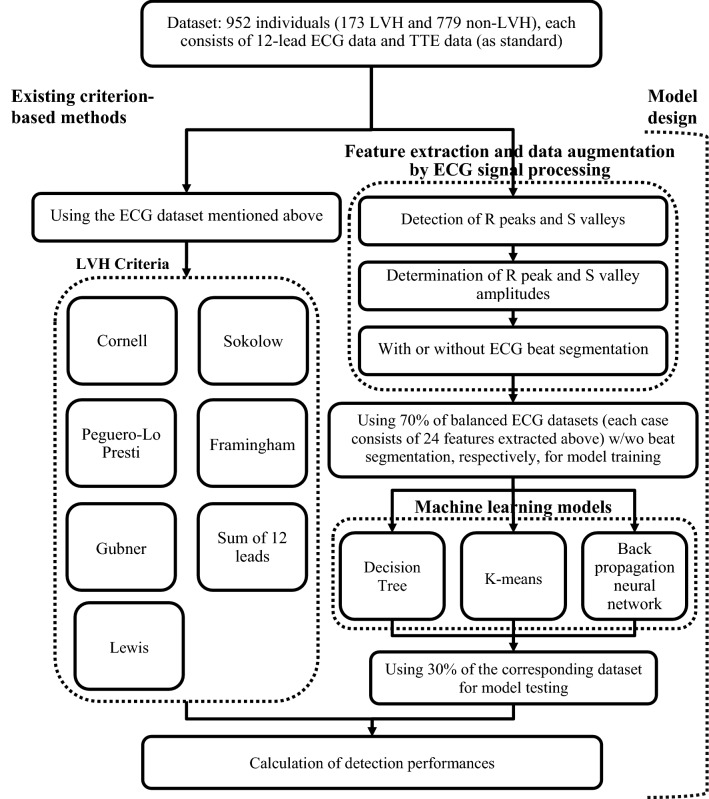
The system architecture of LVH detection.

The system architecture for detecting LVH is illustrated in Fig. 1. Three different machine learning models for detecting LVH with proposed signal processing method (for 12-lead ECG data) were implemented and comprehensively compared to 7 kinds of different criteria. The machine learning models designed in this study adopted 24 features which consisted of R peak and S valley amplitudes automatically detected using the proposed signal processing procedures from 12-lead ECG raw data. Thereby, we hope to improve the overall predictive performance for detecting LVH based on ECG.

### LVH criteria

Different ECG-based criteria have been proposed for assessing LVH. Seven kinds of ECG-based criteria, listed in Table 1, were adopted in this study for comparing their performances to those of machine learning models for detecting LVH.

**Table 1:** Seven methods, ECG-based criteria, for assessing LVH.

Criterion name	ECG criteria for assessing LVH
Cornell^32^	SV3 + RaVL > 2.8 mV (man) SV3 + RaVL > 2.0 mV (woman)
Sokolow^7^	SV1 + max (RV5 or RV6) ≥ 3.5 mV
Peguero^11^	SD (deepest S wave in any lead) + SV4 ≥ 2.8 mV (man) SD (deepest S wave in any lead) + SV4 ≥ 2.3 mV (woman)
Framingham^33^	RaVL > 1.1 mV, RV4 or RV5 or RV6 > 2.5 mV, SV1 or SV2 or SV3 > 2.5 mV, max (SV1 or SV2) + max (RV5 or RV6) > 3.5 mV, or RI + SIII > 2.5 mV
Gubner^34^	RI + SIII ≥ 2.2 mV
Sum of 12 leads^35^	Sum of max (R, S) amplitude in each of the 12 lead ≥ 17.9 mV
Lewis^36^	(RI + SIII) − (RIII + SI) > 1.7 mV

### ECG signal processing

#### Detection of R peaks and S valleys

In order to establish a machine-learning model for the LVH detection system, the first step was to ensure that all the R peaks and S valleys of an ECG sequence could be extracted automatically. In wave detection methods of ECG signals, the R peak is often detected first, and then the relative positions and characteristics of other waves are used to detect other wave peaks and valleys^37–41^. For example, an S valley is usually the first lowest voltage point following a R peak. In this study, a Philips ECG machine was used to measure the ECG signal. Its sampling frequency was 500 Hz and sampling period was 11 s, hence 5500 sampling points of each lead were acquired for each case. MATLAB (Mathwork Inc.) was used to read and process the ECG signals.

Let *X* = [*x*_*1*_, *x*_*2*_, *x*_*3*_,…, *x*_*5500*_] be the raw data of each ECG sequence; *R*_*i*_ is the peak (highest point) of the *i*th R wave in X, *S*_*i*_ is the valley (lowest point) of the *i*th S wave in *X*, *mpa* is the minimum peak amplitude of all R waves, and *miR* is the minimum interval between two adjacent R peaks. Our algorithm for detecting R peaks and S valleys is summarized as follows.

1. Set *mpa* = 0.025 mV
2. Set *miR* = 300 (This is the number of points and stands for 0.6 s)
3. Search for R peaks from the starting point *x*_*1*_ of ECG data sequence by the following conditions:
  - *x*_*j*_ > *mpa*
  - (*x*_*j-1*_ < *x*_*j*_) and (*x*_*j*+*1*_ < *x*_*j*_)

If point *x*_*j*_ meets conditions (a) and (b), then it is set as *R*_*i*_

4. Search for the next peak *R*_*i*+*1*_ from potential points with index ≥ (*j* + *miR*) and meet two criteria (a) and (b) described in step 3
5. Repeat step 4 until the data index (*j* + *miR*) > the end index (= 5500 here)
6. Search for the minimum amplitude within 0.12 s forward from each *R*_*i*_ and set the point as *S*_*i*_
7. Search for the minimum amplitude *m* backward within 0.25 s from each *R*_*i*_. If *m* < *S*_*i*_, then update *S*_*i*_ as *m*, and search for the maximum amplitude within 0.12 forward from the updated *S*_*i*_ and update that point as *R*_*i*_
8. Repeat step 7 for each R peak preliminarily detected in step 5

The above steps are interpreted as follows. First, the function of setting the minimum peak amplitude, *mpa*, is to limit the minimum amplitude of an R wave peak to be captured to filter out the peaks with too small amplitudes in the ECG signal. And *mpa* = 0.025 mV was set in this study. Secondly, the heart rate of a human is limited (normally within 60–100 ppm, i.e. 1–0.6 s per heart beat). the minimum interval between two adjacent R peaks, *miR*, is adopted to limit the time interval of two adjacent R peaks to be detected sequentially and to filter out the next R peak too close to its neighbor. Furthermore, the R peak is often a local maximum and conditions defined by *mpa* and *miR* (steps 3–5) were criteria adopted to preliminarily detect R peaks in this study.

Moreover, the S valley usually appears within 0.12 s following a R peak^42^, and this characteristic can be applied in detecting S valley after detecting each R peak (step 6). However, in the ECG sometimes the peak amplitude of T wave is greater than the peak amplitude of R wave, which will result in the incorrect detection of some T peaks as R peaks. In order to avoid this situation, the algorithm will check that if there is a point with lower amplitude than this preliminarily detected S valley within 0.25 s period before each preliminarily detected R peak. If there is, then the point will replace the preliminarily detected S valley. After confirming the S valley, the R peak will be searched for the point with maximum amplitude within 0.12 s interval before this S valley. Then the preliminarily detected R peak will be replaced by this point (step 7). This step will be repeated for all R peaks detected preliminarily (step 8). Then the algorithm for automatically detecting R peaks and S valleys will be finished.

#### Determination of R peak and S valley amplitudes

In general, there were 8–13 ECG cycles per ECG signal sequence (which resulted from differences in heart rate and that some artifacts or small amplitude cycles might occur in some leads during ECG measurement) retrieved in this study after the detection of R peaks and S valleys. Since the first and last cycles might be incomplete or noisy, they were removed first. Then the median amplitudes of detected R peaks and S valleys from the rest ECG cycles were obtained as the R amplitude and S amplitude for each lead. This processing may make the automatically computed R and S amplitudes closer to the actual values measured manually by the physician.

#### ECG beat segmentation

Only ECG data of 173 LVH cases existed in the original dataset, which were few for designing a machine-learning model. Therefore, the beat segmentation method, Pan-Tompkins technique proposed in^43^, was performed in this study to increase the ECG data amount to improve the detection performances. Detected R peaks were used as distinctive points in the beat segmentation method. Similarly, the first and last cycles were both excluded for each ECG sequence to avoid increasing the training and testing errors of machine-learning model for LVH detection described in the following section. After ECG beat segmentation, we obtained 8.47-fold beats in average in this study.

### Machine learning models for LVH detection

Three machine-learning algorithms, decision tree, k-means, and back propagation neural network, were implemented with Python and used for LVH detection in this study.

#### Decision tree

A decision tree (DT), a supervised machine-learning method, is suitable for classification and regression applications of data science^44^ and features the intuitive result and short execution time. Compared with other machine-learning methods, each decision-making stage of the decision tree is very clear and easily to be understood.

In order to prevent endless growth of the decision tree during classification and result in overfitting, the depth of the decision tree is limited within 1–25. The tree with the highest accuracy is selected as the desired model. The k-fold cross validation^45,46^ is often used to train a machine learning model and validate the predictive performance in the phase of model training. In this study, tenfold cross validation was used to evaluate the model accuracy of each decision tree with a certain depth. The *Entropy*, shown in Eq. (1), is used to assess the performance of each classification node of the decision tree. In a binary classification problem, *p* is the ratio of positive cases in a node and *q* is the ratio of negative cases in the same node. When all the cases classified to a node are exactly the same type, then the *Entropy* = 0. It means an ideal classification. However, if a half of the cases classified to a node are positive and the other half are negative, then the *Entropy* = 1. It means the most non-ideal classification.

1 $$Entropy = p \cdot log_{2} p - q \cdot log_{2} q$$

#### K-means

The k-means algorithm, which is quite often used for clustering^47^, can automatically determine each data point should belong to which category by continuously calculating the distance between two data points. The k-means algorithm features the simple concept and high computational efficiency (even used for clustering a large amount of data). The k value in the algorithm is preset. Initial group centers in the k-means algorithm often have a significant impact on the classification results and the k-means++ algorithm^48^, a modified k-means algorithm which is based on maximizing the distance between the initial group centers, was adopted in this study.

#### Back propagation neural network

**Figure 2:**
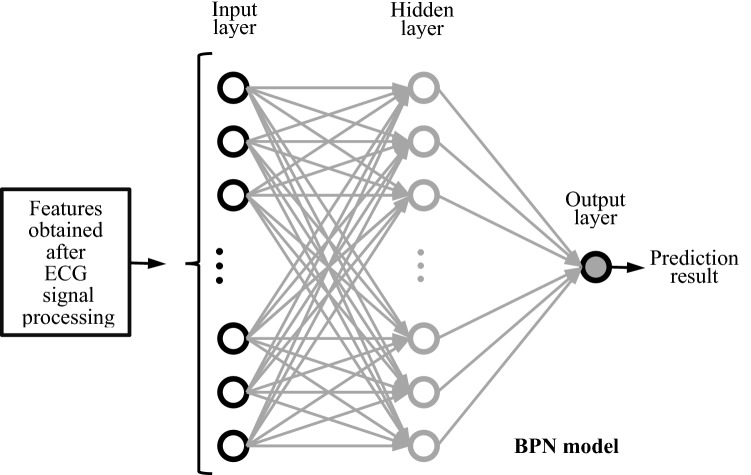
The structure of back propagation neural network (BPN).

The back propagation neural network (BPN) is one of methods developed to imitate biological neural networks^49^. The structure of BPN, illustrated in Fig. 2, is a feedforward neural network with multilayer structure and trained by error back propagation algorithm^50^. Recently, BPN still remains one of the popular machine learning methods or an important base for different AI applications, including predicting or classifying many health and medical events^50–55^, such as BPN associated models for predicting medical expenses and all-cause risk of 30-day readmission, detecting of COVID-19 disease and ischemic stroke, diagnosing hypertrophic cardiomyopathy and hypertensive heart disease, classifying arrythmia disease (with ECG signals), etc.

A neural network can also be regarded as a nonlinear statistical technique with a great learning ability for creating association between input nodes and output nodes of a certain application. The basic architecture of the BPN consists of three parts, namely, the input layer, hidden layer and output layer. The input layer of our BPN model adopted 24 nodes which corresponds to 24 features extracted automatically by ECG signal processing method with 3 procedures (described in section "ECG signal processing" and illustrated in Fig. 1) implemented using the MATLAB program from 12-lead ECG data. These 24 features were amplitudes of R peaks and S valleys of 12-lead ECG signals and were input characteristics of 3 LVH-detection models designed in this study. There is still no good design rule for determining the number of hidden layers and number of nodes of each hidden layer of a neural network for obtaining the best predictive performances^45,46^. After performing a lot of experiments and examining their detection performances, we adopted 1 hidden layer with 26 nodes for the hidden layer structure of BPN model. And the output layer indicates with or without LVH.

The z-score normalization^56^ was adopted before model training, which normalized the mean and standard deviation of data for each feature to be 0 and 1, respectively. Tenfold cross validation^46^ and epoch = 400 were adopt for the BPN model training.

### *F*-statistic for feature importance evaluation

Many features obtained from 12-lead ECG signals can be included in model design for detecting LVH. Moreover, the feature importance between LVH and non-LVH groups can be obtained and important features can be ranked by using *F*-statistic values of all features^57,58^. The *F*-statistic was used to evaluate the importance of all features adopted in creating the BPN model for LVH detection in this study and it is defined as Eq. (2):

2 $$F = \frac{{\sum\nolimits_{g = 1}^{G} {n_{g} (\overline{f}_{g} - \overline{f})^{2} /(G - 1)} }}{{\sum\nolimits_{g = 1}^{G} {\sum\nolimits_{h = 1}^{{n_{g} }} {(f_{g}^{h} - \overline{f}_{g} )^{2} /(n - G)} } }}$$

where *n* is the number of samples (each sample with *p* features), *G* is the number of total groups, *n*_*g*_ is the number of samples in *g*-th group, $\overline{f}_{g}$ is the mean of *g*-th group, $\overline{f}$ is the mean of all samples, and $f_{g}^{h}$ is the feature value of *h*-th sample in *g*-th group. The between-group and within-group variances are included in the numerator and denominator of Eq. (2), respectively. The large *F*-statistic value of a feature indicate a significant difference between the LVH and non-LVH groups.

## Experimental results

### Detection validation of R-peaks and S-valleys

In order to verify the effect of the algorithm on detecting R peaks and S valleys (described in section "ECG signal processing"), mean-square errors (MSEs) and Pearson correlation coefficients between the detected and actual amplitudes of R peaks and S valleys in each sequence of 12-lead ECG signals of randomly selected 30 individuals were calculated and listed in Table 2. It shows the great effect (low MSEs and high correlation coefficients) of the algorithm on detecting R peaks and S valleys.

**Table 2:** MSEs and correlation coefficients between detected and actual amplitudes of R peaks and S valleys in each sequence of 12-lead ECG signals of 30 individuals.

Lead	MSE (10^−8^ V^2^)	Correlation coefficient
SII	0.00112	0.991
RaVR	0.00163	0.904
SIII	0.00172	0.977
SaVF	0.00213	0.989
RV3	0.00260	0.997
RI	0.00267	0.988
SI	0.00308	0.965
RaVL	0.00390	0.966
RV6	0.00409	0.995
RV2	0.00515	0.995
RII	0.00520	0.994
SaVL	0.00537	0.986
RV5	0.00548	0.997
RV4	0.00553	0.995
SV5	0.00584	0.975
SV6	0.00598	0.962
RaVF	0.00608	0.992
RV1	0.00673	0.989
RIII	0.00929	0.988
SV4	0.01158	0.947
SV3	0.02237	0.965
SV2	0.03751	0.975
SaVR	0.04108	0.945
SV1	0.05410	0.957

### Performances of ECG criteria for assessing LVH

Performances of 7 kinds of different ECG criteria for assessing LVH, described in section "LVH criteria", performed in this study are listed in Table 3. It can be seen that the accuracy of all criteria was within 0.50–0.62 and was not accurate enough in diagnosing LVH. The precision and specificity of Lewis criterion were both excellent, however, the sensitivity and accuracy of Lewis criterion were both very low. A similar phenomenon was observed in the performance of Cornell criteria.

**Table 3:** Performances of 7 methods with different ECG criteria for assessing LVH.

Criterion name	Accuracy	Precision	Sensitivity	Specificity
Cornell	0.54	0.88	0.09	0.99
Sokolow	0.57	0.69	0.26	0.88
Peguero	0.57	0.60	0.44	0.71
Framingham	**0.62**	0.62	**0.66**	0.59
Gubner	0.50	0.67	0.01	0.99
Sum of 12 leads	**0.62**	0.65	0.53	0.71
Lewis	0.52	**1.00**	0.03	**1.00**

The bold and underlined values indicate the maximum and minimum values.

### LVH detection performances of machine learning models

In this study, two datasets were separately used to design the machine learning models for detecting LVH. One dataset included 12-lead ECG data of 173 LVH and 173 non LVH cases without ECG beat segmentation (section "ECG beat segmentation") and the other dataset included 1466 LVH and 1466 non-LVH data obtained by performing the ECG beat segmentation from 173 LVH cases and 173 non-LVH cases. The case ratio of training data to testing data was 7:3. Detection performances of 3 models in detecting LVH are shown in Table 4.

**Table 4:** LVH detection performances of machine learning models.

Models	Accuracy	Precision	Sensitivity	Specificity
Without ECG beat segmentation				
Decision Tree	0.74	0.85	0.74	0.85
BPN	0.73	0.75	0.72	0.73
K-means	0.51	0.51	0.46	0.56
With ECG beat segmentation				
Decision Tree	0.92	0.94	0.90	0.94
BPN	**0.961**	**0.958**	**0.966**	**0.956**
K-means	0.59	0.56	0.79	0.38

The bold and underlined values indicate the maximum and minimum values, respectively, of each column with ECG beat segmentation.

The corresponding detection performances (accuracy, precision, sensitivity and specificity) of three models were all improved after adopting ECG beat segmentation, except for the specificity of k-means model. The detection performances of BPN model (accuracy = 0.961, precision = 0.958, sensitivity = 0.966 and specificity = 0.956, respectively) are the highest among these 3 models with ECG beat segmentation.

**Figure 3:**
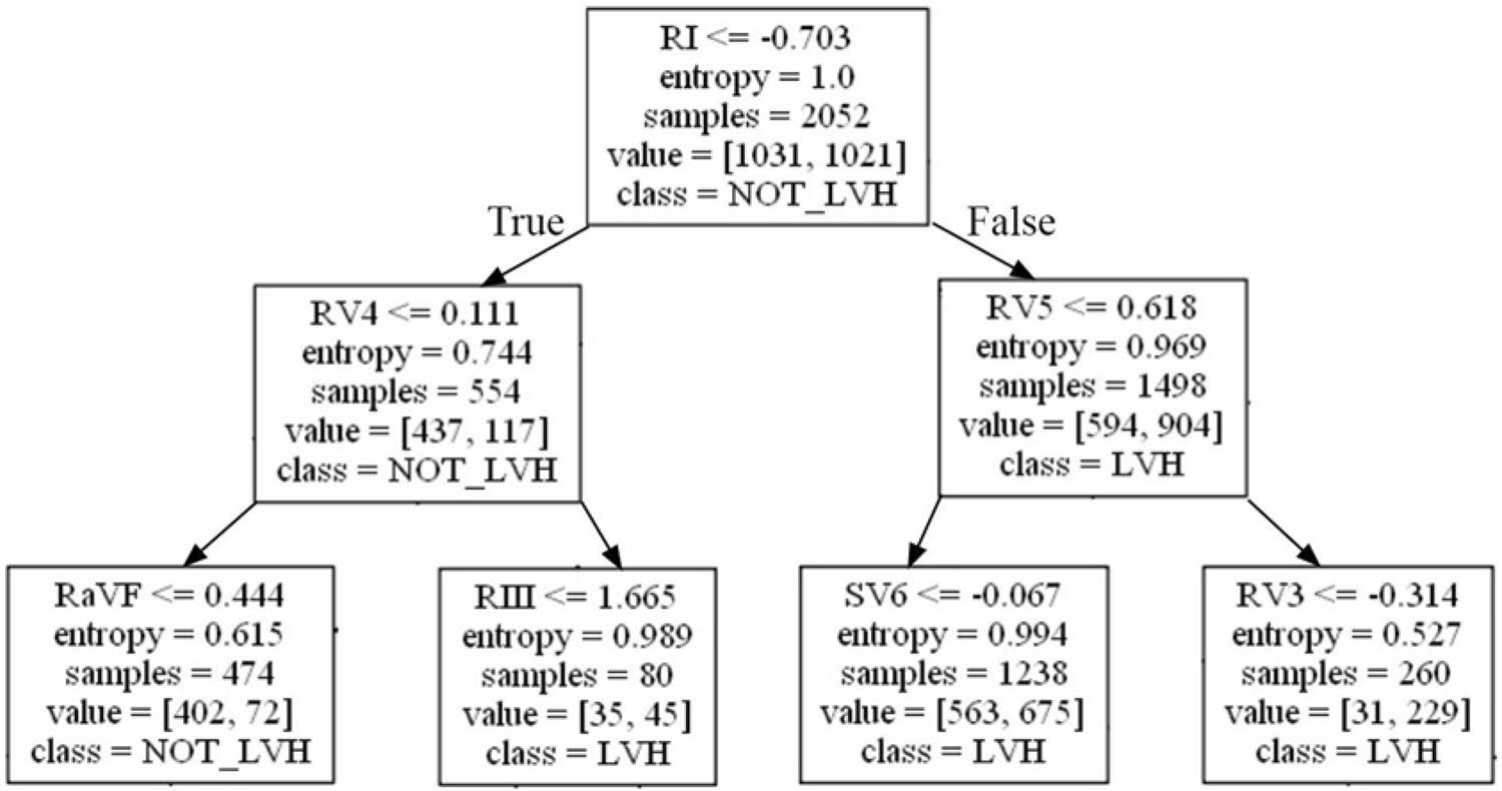
The first three layers of the decision tree after ECG beat segmentation.

The growth situation of our decision tree model, Fig. 3, with ECG beat segmentation shows the readability of a decision tree model for LVH detection as well as the similarity between a decision tree model and the existing ECG criteria for LVH detection. Four-lead signals, adopted in the seven nodes of the top three layers of the decision tree model, are also adopted in 6 of 7 ECG criteria listed in Table 1. These 4-lead signals are RI (also adopted in criteria of Framingham, Gubner and Lewis), RV4 (also adopted in Framingham criteria), RV5 (also adopted in criteria of Sokolow–Lyon and Framingham) and RIII (also adopted in Lewis criterion). Of course, Peguero and sum of 12 leads (two of the 7 criteria listed in Table 1 for assessing LVH) consider all of the 12-lead ECG signals.

### Feature importance

All of the 24 features, amplitudes of R peaks and S valleys, obtained from the output of ECG signal processing method (section "ECG signal processing") were adopted to design machine learning models in the current study. The importance of each feature was evaluated using the *F*-statistic^57,58^, expressed in Eq. (2), and the calculated *F*-statistic values of all features were ranked in Table 5. Five of the top six features (except for RaVF) with the highest *F*-statistic values are also features used by at least one of criterion methods listed in Table 1 (Here, we exclude the method of sum of 12 leads, because it considers all of the 24 features obtained from 12-lead ECG signals).

**Table 5:** *F*-statistic values of adopted features.

Feature	*F*-statistic value
RI	682.6
RaVL	469.5
RIII	151.5
RV5	150.1
RaVF	141.7
RV4	136.5
SaVR	103.7
SV4	78.3
SaVL	67.9
RV6	45.8
SII	45.6
RV3	42.2
SV5	37.7
SaVF	34.6
SI	34.1
SIII	33.4
SV2	26.9
SV3	22.2
RII	15.4
RaVR	12.6
RV2	7.8
RV1	6.8
SV6	3.5
SV1	2.1

**Figure 4:**
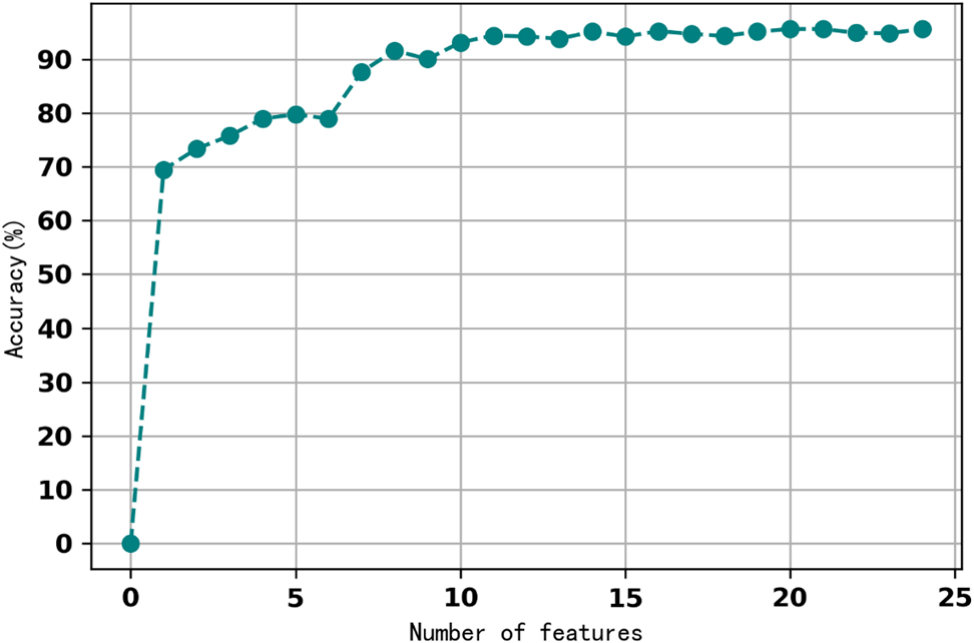
Accuracy variation of the number of features.

According to the *F*-statistics of features, the feature with the lowest *F*-statistic value was excluded sequentially in the training and testing phases for a BPN model. We obtained the accuracy of redesigned BPN model versus adopted number of features as illustrated in Fig. 4. When the number of features was down to seven, the accuracy decreased less than 10%. This indicated that the top seven features listed in Table 5 were most related to LVH.

## Discussions

### Detection performances in the current study

Detection performances of machine learning models and criteria-based methods performed in this study for assessing Left ventricular hypertrophy are discussed as follows. The numbers of considered leads of seven methods listed in Table 1 are 2, 3, 12, 9, 2, 12 and 2, respectively. Four of seven methods of ECG-based criteria consider only few (2 or 3) leads of 12-lead ECG signals in assessing LVH. The other three criteria (Peguero, Framingham, and Sum of 12 leads) consider all or 9 leads in assessing LVH. Basically, inequalities with thresholds are adopted as evaluation criteria. Comparing to the machine learning model, such as BPN adopted in this study, more comprehensive recognition for assessing LVH can be built into the machine learning model during the training phase using all 12-lead ECG signals. Furthermore, we adopted the ECG signal processing method (described in section "ECG signal processing") including an automatic detection of R-peak and S-valley amplitudes and ECG beat segmentation before designing machine learning models in the current study.

The LVH indirectly implies cardiac overloading and it can be diagnosed by ECG such as the voltage criteria^11^. However, the waveform details of multiple ECG cycles of the same LVH subject might differ slightly^11^, and the indirect manifestation level in R peak and S valley of each ECG cycle of a LVH subject might differ slightly. Using only the single-cycle ECG signal to determine if a person is LVH or not, like the seven methods listed in Table 1 often do^11^, may not be comprehensive enough. Above description may be one of the common reasons for low accuracy of ECG criteria (7 methods listed in Table 1) in detecting LVH. Even by averaging the signals of more ECG cycles for each criteria-based method, the relation between R peak and S valley of each cycle may be changed due to the average process.

On the contrary, in this study we tried to utilize comprehensive signals of ECG cycles and the details hiding in each ECG cycle of each LVH subject to design a model for LVH detection. After ECG beat segmentation (section "ECG beat segmentation"), we could adopt more ECG cycles (8.47-fold cycles in average) of each LVH subject, which might contain more information details among these increased ECG cycles (increased 7.47 cycles in average). In other words, after ECG beat segmentation, we could adopt the more comprehensive and detail dataset with 8.47-fold number of ECG cycles (1021 positive and 1031 negative cases) to train the BPN model. Hence, the BPN model could learn more on both what LVH and non-LVH should be from the larger dataset. This in turn improved detection performances of designed BPN model in the test phase. This explains that detection performances of a BPN model designed with a dataset obtained after beat segmentation outperformed the BPN model designed with original dataset (118 positive and 124 negative cases) without beat segmentation as shown in Table 4. Moreover, this also explains detection performances (the penultimate row in Table 4) of the BPN model designed using 12-lead ECG signals with ECG beat segmentation greatly outperformed existing seven methods (Table 3) using criteria defined from fewer leads and a single cycle of ECG signals.

### Comparison of state-of-the-art methods for detecting LVH using ECG

New methods or machine learning models for detecting LVH using ECG signals reported in previous studies are summarized in Table 6. Where the criteria methods based on ECG signals are summarized in the top 7 studies^12,14–18,23^ and described as follows. In Ref.^16^, Sokolow–Lyon, Cornell and Cornell Product (CP) criteria were adopted to detect LVH of elderly Chinese aged > 65 years. CP reached the greatest performance (AUC = 0.62) but it is still unsatisfactory. In Ref.^17^, a combination of 6 methods of ECG criteria was proposed to detect LVH. This combined method reached slightly better detection performances for detecting LVH in a hypertension cohort than a general population. In Ref.^18^, Peguero-Lo Presti criteria outperformed Cornell voltage, Sokolow–Lyon voltage and Romhilt-Estes criteria for detecting LVH of patients ≥ 70 years old and 83.1% with hypertension. Their Peguero-Lo Presti criterion reached precision, sensitivity and specificity (0.665, 0.519, and 0.821, respectively) and were slightly better than our results (0.60, 0.44, and 0.71, respectively) listed in Table 3 (Peguero).

**Table 6:** Comparison of state-of-the-art methods for detecting LVH using ECG.

Study (year)	Method	Adopted features	Detection performances					Limitation
			ACC	PRE	SEN	SPE	Others	
ECG criteria								
Ref.^16^ 2019	Cornell Product criteria	Multi-lead ECG	–	–	–	–	AUC = 0.62	Patients with age < 65 y were excluded
Ref.^17^ 2019	Combined criteria	12-lead ECG	–	0.401	0.379	0.915	AUC = 0.65	For a untreated hypertension cohort
Ref.^18^ 2021	Peguero-Lo Presti criteria	12-lead ECG	–	0.665	0.519	0.821	AUC = 0.7	Patients with age < 70 y were excluded
Ref.^15^ 2021	Peguero–Lo Presti	Multi-lead ECG	0.68	0.12	0.29	0.73	NPV = 0.89	
	Cornell voltage		**0.86**	0.24	0.12	0.95	NPV = 0.89	
	Cornell product		**0.86**	0.12	0.04	**0.96**	NPV = 0.89	
	Sokolow–Lyon voltage		0.81	0.13	0.12	0.89	NPV = 0.89	
	Sokolow–Lyon product		**0.86**	0.13	0.04	**0.96**	NPV = 0.89	
Ref.^12^ 2021	NCRCHS^#1^ criterion with multiple linear regression	3-lead ECG	–	–	**0.90**	0.36	AUC = 0.74	
Ref.^23^ 2021	CHCM^#2^	3-lead ECG	0.705	–	0.743	0.687		
Ref.^14^ 2021	RaVL voltage-duration product	Lead aVL ECG	–	**0.756**	0.674	0.546	AUC = 0.64	In older individuals with left bundle branch block
	Sokolow–Lyon criteria	3-lead ECG	–	0.75	0.261	0.818	AUC = 0.54	
Machine learning models								
Ref.^27^ 2018	Random forest	ECG data	0.661	–	0.58	0.709		
Ref.^20^ 2019	BART^#3^-LVH criteria	26 features^#4^	–	0.299	0.29	0.946	AUC = 0.829	Participants without cardiovascular disease at enrollment
Ref.^21^ 2020	Decision tree with logistic regression	6 ECG features	0.733	–	0.816	0.693		
Ref.^24^ 2020	Deep neural network	87 ECG features	0.736	0.73	0.667	0.782		
Ref.^25^ 2020	Ensemble neural network^#5^	12-lead ECG signals + demographic features^#6^	0.851	–	0.613	0.896	AUC = 0.868	
Ref.^26^ 2021	Convolution neural network	12-lead ECG	–	–	0.96	0.34	AUC = 0.653	
Ref.^22^ 2021	GLMNet^#7^	34-feature 12-lead ECG	–	–	–	–	AUC = 0.873	
This study	BPN	24-feature 12-lead ECG with ECG beat seg-mentation	**0.961**	**0.958**	**0.966**	**0.956**		Participants without arrhythmia

*ACC* Accuracy, *PRE* Precision, *SEN* Sensitivity, *SPE* Specificity, *AUC* area under ROC curve, *NVP* negative predictive value.

^#^^1^NCRCHS = Northeast China Rural Cardiovascular Health Study.

^#^^2^CHCM = Cardiac Hypertrophy Computer-based Model.

^#^^3^BART = Bayesian Additive Regression Trees.

^#4^26 features include age, sex, height, systolic and diastolic blood pressures and 21 ECG features.

^#^^5^Ensemble neural network = convolutional neural network + deep neural network.

^#^^6^demographic features include age, sex, weight and height.

^#^^7^GLMNet = penalized logistic regression with the ElasticNet penalty.

The bold and underlined values indicate the maximum and minimum values, respectively, of each column for ECG criteria or machine learning models.

In Ref.^15^, five methods of ECG criteria (Peguero–Lo Presti, Cornell voltage, Cornell product, Sokolow–Lyon voltage and Sokolow–Lyon product) for LVH detection of a general Chinese population were studied. These ECG-LVH criteria reached negative predictive value (NPV = 0.89), accuracy (0.68–0.86), specificity (0.73–0.96), positive predictive value (0.12–0.24) and sensitivity (0.04–0.29). They concluded that these 5 ECG-LVH criteria had high NPV to detect Echo-LVH. Three of the 5 methods were also adopted in our current study. In Ref.^12^, the NCRCHS (Northeast China Rural Cardiovascular Health Study) criterion, constructed with multiple linear regression to assess the relationship between ECG-LVH criteria and LVMI by using 3-lead ECG signals, was proposed in 2021. In Ref.^23^, cardiac hypertrophy computer-based model (CHCM) was proposed to detect LVH using criteria of the T voltage in lead I (≤ 0.055 mV), peak-to-peak QRS distance in lead aVL (> 1.235 mV), and peak-to-peak QRS distance in lead aVF (> 0.178 mV). In Ref.^14^, 10 methods of ECG criteria were used to detect LVH in an elder cohort. Two of the methods (RaVL voltage-duration product and Sokolow–Lyon criteria) with the best detection performances are listed in Table 6.

Above methods of ECG criteria (listed in Table 6) reached detection performances of accuracy, precision, sensitivity and specificity within 0.68–0.86, 0.12–0.756, 0.04–0.9, and 0.36–0.96, respectively. The precision, sensitivity and specificity among these studies ranged widely. Our prediction performances of 7 methods of criteria listed in Table 3 reached the accuracy, precision, sensitivity and specificity within 0.50–0.62, 0.60–1.0, 0.01–0.66, and 0.59–1, respectively. Similarly, the precision, sensitivity and specificity among our results obtained using 7 methods of criteria ranged widely. The best precision and specificity in our results outperform previous studies^12,14–18,23^, while the best accuracy and sensitivity of ours are lower than some of these studies. Unfortunately, the detection performances listed in Tables 3 and 6 show that all these methods of ECG criteria are not perfect enough in detecting LVH by evaluating using the accuracy, precision, sensitivity and specificity.

On the other hand, machine learning models for detecting LVH using ECG signals reported in previous studies^20–22,24–27^) are also summarized in Table 6. In Ref.^27^, the random forest model was designed using ECG data with K-nearest neighbor and Z-score for LVH detection. In Ref.^20^, the BART-LVH criteria constructed using Bayesian additive regression trees with 26 features (include age, sex, height, systolic and diastolic blood pressures and 21 ECG features) was proposed for LVH detection. In Ref.^21^, a decision tree model with logistic regression for dimensionality reduction using 6 features of ECG signals was proposed for detecting LVH.

In Ref.^24^, a model of deep neural network (DNN) with 6 layers was trained using 87 ECG features and developed for detecting LVH. In Ref.^25^, a model of ensemble neural network (ENN) which integrated the convolution neural network (CNN) and DNN was proposed in predicting LVH. The 8-s 12-lead ECG signals (two-dimensional data, 4000 × 12) were input to the CNN, while ECG features and demographic features (age, sex, weight and height) were input to the DNN. Then the outputs of CNN and DNN were integrated together to generate an output of LVH detection.

In Ref.^26^, a CNN was trained with the entire 10-s 12-lead ECG waveform to predict the left ventricular (LV) mass. The cardiac magnetic resonance (CMR)-derived LV mass was used as the standard. Then their CNN model can predict LVH indirectly. In Ref.^22^, several LVH detection models designed using 4 machine learning methods were reported in 2021. In which, the GLMNet (penalized logistic regression with the ElasticNet penalty) model reached the best detection performance with AUC = 0.873. The GLMNet model was designed using 34 features, which include 24 features of R peaks and S valleys obtained from 12-lead ECG signals and 10 more features retrieved from ECG signals.

The detection performances of the aforementioned methods for detecting LVH proposed in previous studies are summarized in Table 6. Above machine learning models proposed in previous studies reached detection performances of accuracy, precision, sensitivity and specificity within 0.661–0.851, 0.299–0.73, 0.29–0.96, and 0.34–0.946, respectively. The precision, sensitivity and specificity of these models designed in previously studies ranged widely. Our prediction performances of BPN model reached the accuracy, precision, sensitivity and specificity equaling 0.961, 0.958, 0.966, and 0.956, respectively. Our BPN model outperforms 7 models, reported in previous studies^20–22,24–27^ and listed in Table 6, in detecting LVH using ECG signals.

Furthermore, in Refs.^12,20–22,24–26^, they concluded that machine learning methods outperform some methods of classical ECG criteria in detecting LVH. Basically, our BPN model also outperforms the methods of criteria reported in previous studies listed in Table 6 in terms of accuracy, precision, sensitivity and specificity. Moreover, Our BPN model mostly obtained the higher and more uniform accuracy, precision, sensitivity and specificity. This might result from that (1) we designed our BPN model using a balanced dataset and which may reduce the difference between sensitivity and specificity of predictive performances of a model^46,59^; (2) we preprocessed 12-lead ECG to automatically and precisely obtain amplitudes of R peaks and S valleys; and (3) we adopted ECG beat segmentation to utilize as many ECG cycles (8.47 cycles per sequence in average) as possible to train our BPN model, such that the model can learn and recognize slight differences among different ECG cycles of each subject.

### Study limitations and future perspectives

The ECG signals retrieved in the current study were all obtained from participants without arrhythmia (e.g. atrial fibrillation), and the algorithm we adopted for automatic detection R peaks and S valleys described in section "Detection of R peaks and S valleys" could limit the heart rate within 60–100 ppm. The modified detection algorithm can be adopted or created for a wider range of heart rates resulting from including participants with arrhythmia in the future study.

Hopefully, our outcome could benefit the accurate detection of LVH or other cardiovascular diseases using ECG and AI. The more comprehensive detection of P, Q, R, S and T points in each ECG wave for obtaining more features (such as the change of ST segment, etc.) automatically for more accurately detecting the LVH or other cardiovascular diseases using an AI method may be considered. So that an AI system (or called clinical decision support system, CDSS^46,59^ for LVH detection, which features accurate, automatic and fast, can be built for supporting diagnosis and providing useful information for physicians.

An AI system may help cardiologists to manage the large demand for ECG, especially when mass screening of hundreds of apparently healthy individuals by ECG is required daily. Our future perspective further includes analyzing other surrogate end points in cardiovascular diseases (such as left ventricular diastolic dysfunction, new-onset myocardial infarction or heart failure, and cardiac-related mortality^31,60^, thereby creating the corresponding AI models for detecting these cardiovascular events accurately.

## Conclusions

We conducted a simple and useful preprocessing method for RS amplitude detection and ECG beat segmentation to obtain 24 features from 12-lead ECG for designing the machine-learning model. Thereby, the designed BPN model might reach the excellent LVH prediction performance and outperform previous reported models and criteria.

## Data Availability

The datasets used and/or analysed during the current study would be available from the corresponding author on reasonable request.
